# Phosphonic acid: preparation and applications

**DOI:** 10.3762/bjoc.13.219

**Published:** 2017-10-20

**Authors:** Charlotte M Sevrain, Mathieu Berchel, Hélène Couthon, Paul-Alain Jaffrès

**Affiliations:** 1 CEMCA UMR CNRS 6521, Université de Brest, IBSAM. 6 Avenue Victor Le Gorgeu, 29238 Brest, France

**Keywords:** bromotrimethylsilane, hydrolysis, McKenna’s reaction, phosphonate, phosphonic acid

## Abstract

The phosphonic acid functional group, which is characterized by a phosphorus atom bonded to three oxygen atoms (two hydroxy groups and one P=O double bond) and one carbon atom, is employed for many applications due to its structural analogy with the phosphate moiety or to its coordination or supramolecular properties. Phosphonic acids were used for their bioactive properties (drug, pro-drug), for bone targeting, for the design of supramolecular or hybrid materials, for the functionalization of surfaces, for analytical purposes, for medical imaging or as phosphoantigen. These applications are covering a large panel of research fields including chemistry, biology and physics thus making the synthesis of phosphonic acids a determinant question for numerous research projects. This review gives, first, an overview of the different fields of application of phosphonic acids that are illustrated with studies mainly selected over the last 20 years. Further, this review reports the different methods that can be used for the synthesis of phosphonic acids from dialkyl or diaryl phosphonate, from dichlorophosphine or dichlorophosphine oxide, from phosphonodiamide, or by oxidation of phosphinic acid. Direct methods that make use of phosphorous acid (H_3_PO_3_) and that produce a phosphonic acid functional group simultaneously to the formation of the P–C bond, are also surveyed. Among all these methods, the dealkylation of dialkyl phosphonates under either acidic conditions (HCl) or using the McKenna procedure (a two-step reaction that makes use of bromotrimethylsilane followed by methanolysis) constitute the best methods to prepare phosphonic acids.

## Review

### 1. Introduction

**Figure 1:**
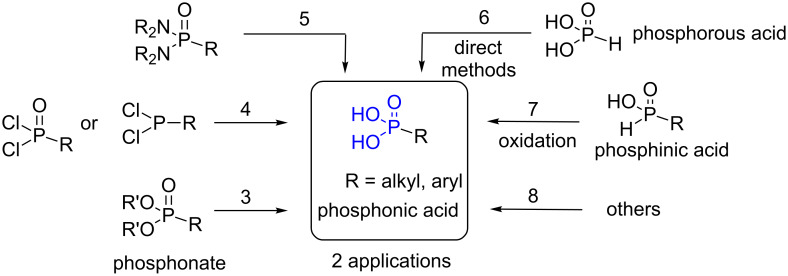
Summary of the synthetic routes to prepare phosphonic acids detailed in this review. The numbers indicate the corresponding sections of this review.

Phosphonic acid is a functional group featuring two hydroxy moieties, one P=O double bond and one P–C bond. This functional group was incorporated in a broad diversity of molecules and polymers to introduce specific properties including water solubility, coordination or supramolecular properties. Several books, book chapters or reviews have been focused on the construction of the P–C bond [1], on the description of the different classes of phosphorus-containing functional groups [2], on specific applications (hybrid materials [3], surface modification [4], oil industry [5]) or dedicated to a family of compounds (e.g., aminophosphonic acids [6], organometallic phosphonic acids [7]). However, no recent review was focused on the different methods that can be employed to prepare phosphonic acids which is a function needed to address numerous applications that are summarized in the first part of this review (section 2). Then, we review the principal methods that can be employed to prepare phosphonic acids. The most frequently applied methods start from phosphonates (section 3 and Figure 1). However, other possibilities exist: the hydrolysis of dichlorophosphine or dichlorophosphine oxide (section 4), the hydrolysis of phosphonodiamide (section 5), the direct methods that make use of phosphorous acid (H_3_PO_3_) to create the P–C bond simultaneously to the formation of phosphonic acid (section 6) or the oxidation of phosphinic acid (section 7). The last section (section 8) includes additional miscellaneous methods to prepare phosphonic acids. Of note, the biosynthesis of phosphonic acid, which is a dynamic field of research aiming to discover new bioactive compounds [8], will not be detailed in this review which is focused on the chemical way of producing phosphonic acids. Of note, this review is not an exhaustive list of all the phosphonic acids synthesized over the last 20 years but a selection of examples that aim to illustrate the most relevant methods that can be employed to produce phosphonic acids.

### 2. Phosphonic acids: properties and applications

**Figure 2:**
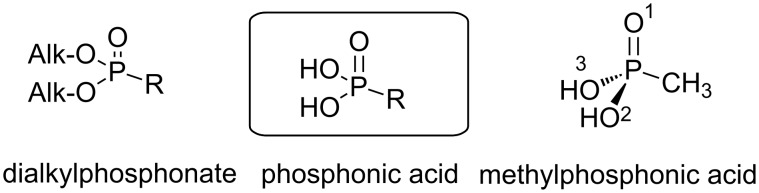
Chemical structure of dialkyl phosphonate, phosphonic acid and illustration of the simplest phosphonic acid: methylphosphonic acid.

In the solid state, a phosphonic acid function possesses one P–O bond which is shorter than the two others and that can be attributed to the P=O double bond (as an example for methylphosphonic acid, Figure 2, the P=O bond length is 1.4993(11) Å, the two other P–O bond lengths are 1.5441(11) Å and 1.5443(12) Å and the P–C bond is 1.7586(17) Å). The bond angles at the phosphorus atom are ranging from 103.46(8)° to 112.86(7)° for methylphosphonic acid, indicating a distorted tetrahedral geometry around the phosphorus [9]. This function is very stable but under some oxidative conditions (e.g., Mn(II) with O_2_) the rupture of the P–C bond can occur to produce phosphate [10]. The phosphonic acid function possesses two acidic protons featuring, for instance, when R is an aromatic moiety, a first p*K*a ranging from 1.1 to 2.3 and the second acidity which features a p*K*_a_ ranging from 5.3 to 7.2 [11]. The values strongly depend on the electronic properties induced by the substituent R. It must be noted that due to the high polarity of the phosphonic acid function, the purification of phosphonic acids is quite difficult except by recrystallization for the solid samples. The purification by chromatography on silica gel requires very polar eluents (e.g., CHCl_3_/MeOH/H_2_O 5:4:1, v/v/v) [12] and further purification by HPLC with a C18 grafted column. Due to these difficulties of purification, it must be noted that in many cases the purification occurs on the precursors of the phosphonic acid. As an example, dialkyl phosphonates (the diester derivatives of phosphonic acid) can be easily purified by chromatography on silica gel and diverse clean and efficient methods can be applied to produce phosphonic acid from phosphonates without the need of intense purification in the final step. Indeed, the purification is limited to remove the solvent and volatile reagents that are frequently used in excess (see section 3). Due to the acidity of phosphonic acid this function is deprotonated in water and this property was used to increase the water solubility of organic compounds [13–14], polymers [15–16] or ligands for coordination chemistry [17]. It must be noted that for similar compounds, that only differ by the replacement of a carboxylic acid group with a phosphonic acid moiety, the log P_OW_ values (partition coefficient between octanol/water solution) are decreased by about 1 log unit (meaning that the phosphonic acid derivatives when placed in an octanol/water mixture the compound’s concentration in the aqueous phase is 10 times the concentration of the corresponding carboxylic acid analogue) [11]. Meanwhile, the phosphonic acid derivatives are more acidic when compared to their carboxylic acid equivalents (1.9 to 2.9 units of p*K*_a_ below for the first acidity of phosphonic acid derivatives) [11]. This property was used to design Brönsted acid catalysts that were used for the depolymerization of cellulose [18] and the synthesis of dihydropyrimidine derivatives [19]. The monosodium salt of phosphonic acids were also employed as organocatalysts for Michael addition [20]. The capacity of phosphonic acids to increase the solubility of organic compounds in water was employed to develop water soluble catalysts [21], or to improve the water solubility of drug-chelate supramolecular assemblies (e.g., calixarene) [22–23]. The water solubility of phosphonic acid is strongly improved when the phosphonic acid is deprotonated (basic media). It is not rare to observe, when preparing NMR tubes with D_2_O as solvent that phosphonic acid appeared weakly soluble whereas the addition of K_2_CO_3_ induced its solubilization. Interestingly the increase of the number of phosphonic acid functional groups on a molecule is not systematically associated with an increase of its water solubility as exemplified with triphenylphosphine functionalized either by two or three phosphonic acid sodium salts; the former being more water soluble than the later [24]. This behavior is associated to the fact that the trisphosphonic acid sodium salt was already solvated at the solid state.

The high polarity of the phosphonic acid function and its ability to be ionized in water render this group attractive to design the polar head group of anionic amphiphilic compounds [25–26] or polymeric-based amphiphilic derivatives [27]. This type of compound was used as surfactant to stabilize colloidal solutions of nanocrystals [28], to prepare oil/water emulsions [29], or to prevent corrosion [30–32]. The ionic interactions between phosphonic acid and lipophilic amine produced catanionic supramolecular aggregates that were assessed as HIV inhibitors [26,33].

**Figure 3:**
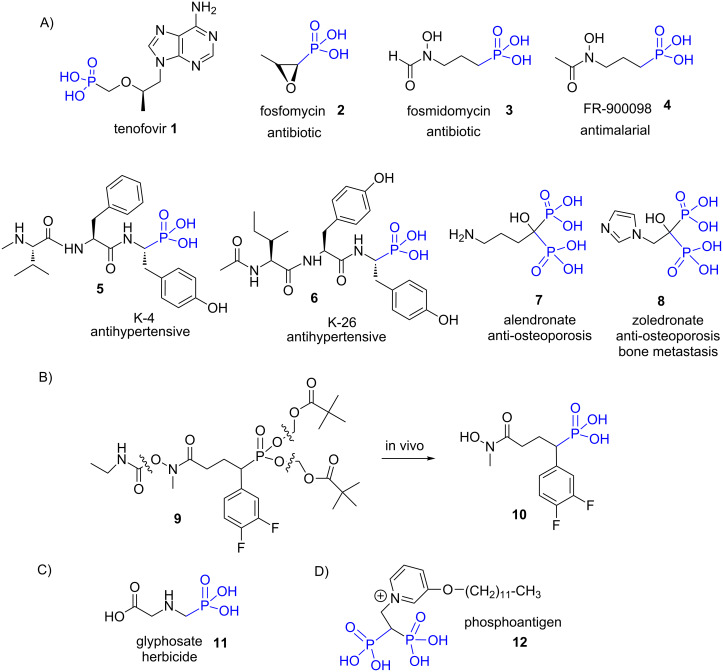
Illustration of some phosphonic acid exhibiting bioactive properties. A) Phosphonic acids for biomedical applications ; B) phosphonic acid generated in vivo from a phosphonate pro-drug; C) chemical structure of glyphosate known for its herbicidal properties; D) chemical structure of a phosphoantigen containing phosphonic acid functional groups.

Many phosphonic acids are present in nature and their biosynthesis involves, as one of the key steps, the isomerization of phosphoenolpyruvate to phosphonopyruvate which is catalyzed by phosphopyruvate mutase [34]. Recent studies indicate that up to 25% of the phosphorus available in the ocean would consist of phosphorus species featuring one P–C bond [35] pointing out the importance of such type of compounds in natural biochemical processes [36]. Phosphonic acid mimics the phosphate group, which is omnipresent in nature, but also the tetrahedral transition-state intermediate encountered for instance during the hydratation of carbon dioxide by carbonic anhydrase [37] or during the hydrolysis of amide [38]. The main difference between phosphate and phosphonic acid arises from the higher stability (resistance towards enzymatic degradation) of the P–C bond when compared to the P–O bond present in phosphate. Accordingly, phosphonic acid was used for numerous applications in biology and medicine to mimic the phosphate group leading to antiretroviral drugs (e.g., tenofovir (**1**)) [39], isoprenoid biosynthesis inhibitors [40–41], antibiotics (e.g., fosfomycin (**2**)) [42], tyrosine phosphatase inhibitors **3** [43], antimalarial **4** [44], antihypertensive drugs (e.g., K4 **5** and K26 **6**) [45] or the anti-osteoporosis compounds alendronate (**7**) [46] and zoledronate (**8**) [47] (Figure 3A). In some cases, the bio-active phosphonic acid is generated in vivo from a phosphonate pro-drug [48] as exemplified by the formation of **10** from **9** which permits to improve the pharmacokinetic properties (Figure 3B) [49]. Some phosphonic acid-containing compounds were also used for their herbicidal properties as exemplified by glyphosate (**11**) [50] (Figure 3C). Finally, in the field of immunotherapy, γδ T cells, at the difference to αβ T cells, respond to non-peptidic antigen or to the alterations in the expression of normal cell surface components. The subset of the human γδ T cells that express the Vγ9Vδ2 receptor recognize and present non-peptide antigens including prenyl pyrophosphate antigens also identified as phosphoantigens. These Vγ9Vδ2 T cells are involved during bacterial or protozoan infections but play also a role in tumor immunity [51]. The synthesis of prenyl pyrophosphate analogues was assessed as a strategy to activate Vγ9Vδ2 T cells [52]. These analogues are mainly composed of pyrophosphate compounds but some phosphonic acids such as compound **12** was identified as an activator of Vγ9Vδ2 T cells [53–54] (Figure 3D).

**Figure 4:**
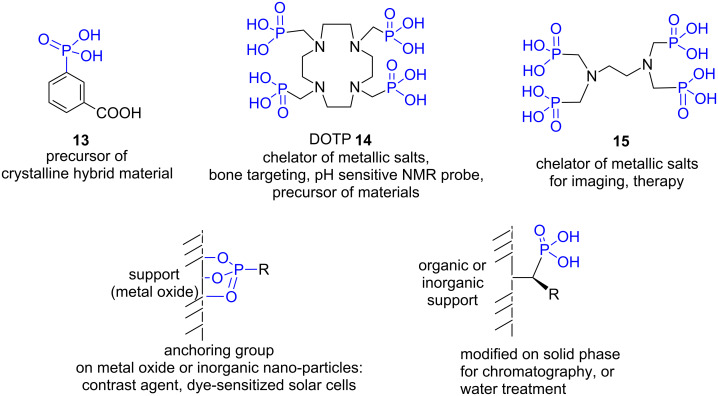
Illustration of the use of phosphonic acids for their coordination properties and their ability to be involved for the synthesis of hybrid materials or to interact with metal-oxide surfaces.

Phosphonic acid is a function that is readily involved in hydrogen bonds thus generating auto-assembling supramolecular structures [55] or, when a second organic partner possessing basic groups is present (e.g., amine), mixt supramolecular materials [56–58]. This supramolecular behavior was also involved in the development of organic materials exhibiting proton conduction [59], or organo-gel properties [60]. In the field of analytical chemistry, molecules functionalized with phosphonic acid groups were used to prepare the solid phase for immobilized metal affinity chromatography (IMAC). Such types of solid phases were applied to enrich carbohydrate from extracts [61] or as chiral selectors immobilized on silica for chiral cation exchange chromatography [62] (Figure 4). Porphyrin tetra-functionalized with phosphonic acid groups was used as a chemosensor for trinitrotoluene (TNT) [63].

The third important feature of phosphonic acid arises from its coordination properties. Indeed, the three oxygen atoms can be engaged in coordination or iono-covalent bonds. These coordination properties were used to design molecular hybrid materials [64] also identified as metal organic framework (MOF) or coordination polymers that are synthesized by reaction with a metallic salts (e.g., copper [65], lanthanides [66]) under hydrothermal conditions [67–68] or by insertion of phosphonic acid in low dimensional inorganic materials like layered double hydroxide (LDH) [69] or layered simple hydroxide (LSH) [70]. These materials prepared from phosphonic acids were assessed for numerus applications, including nuclear fuel stewardship and separations of actinides [71], porosity [72–74], bactericidal properties [75], inorganic salt release [76], luminescence [77–78], protonic conduction [79–80], materials with magnetic properties [81–82], or were spread off in polymers to produce nanocomposites [83–84]. Of note, despite the non-chiral nature of compound **13** (Figure 4), it produced, when associated with copper salt, a homochiral non-centrosymmetric crystalline hybrid material [85]. The coordination properties of phosphonic acid were also applied to design tetraazamacrocyclic compounds functionalized with phosphonic acid pendant arms. As an example, ((1,4,7,10-tetraazacyclododecane-1,4,7,10-tetrayl)tetrakis(methylene))tetraphosphonic acid, DOTP, compound **14**, Figure 4), was assessed to complex indium [86] or terbium [87] with the aim to develop bone targeting and dosimetry. It was also employed to complex ^89^Y and applied as pH sensitive NMR probe [88], or as organic precursor of crystalline manganese, nickel [89] or lanthanide-containing hybrid materials [90]. Such type of chelating agents was also designed to develop responsive contrast agents for magnetic resonance spectroscopy [91]. Acyclic diamine compounds (e.g., **15**, Figure 4) possessing phosphonic acid groups as additional coordination sites or the tetraazamacrocyclic compound **14** were also reported as a chelator of ^177^Lu which was explored as a radiotracer that accumulates in bones [92]. These coordination properties were also applied to water treatment and to selectively extract lanthanide from water solutions [93], for nuclear waste treatment [94] or as sequestration or decorporation agents [95]. The high affinity of phosphonic acid to calcium ions is another feature of the coordination properties of phosphonic acids that triggered biomedical applications. This coordination property for calcium cations was further enhanced by placing two phosphonic acid moieties and one hydroxy group around a methylene unit (hydroxymethylenediphosphonic acid). Such type of molecular fragment was incorporated in the structure of drugs used to reduce bone resorption that occurs in bone metastastis or osteoporosis (alendronate (**7**), zoledranote (**8**), Figure 3). This affinity for calcium was also used to develop bone targeting which was assessed for therapy [96] and imaging [97–98]. Finally, the phosphonic acid group was also employed to immobilize organic or organometallic molecules on the surface of metal oxide [99–100] (Al_2_O_3_ [101], TiO_2_ [102–103], SnO_2_ [104], Fe_3_O_4_ [105], ZnO [106]), CdSe quantum dots [107] and used as anchoring group for dye-sensitized solar cells [108–110] or for the immobilization of organocatalyst [111–112]. Phosphonic acid was also used for coating superparamagnetic iron oxide (e.g., magnetite) assessed as contrast agent in magnetic resonance imaging [113] or to conceive a red/ox catalyst that can be magnetically separated from the reaction media [114]. The immobilization of phosphonic acid can be also achieved by supramolecular interaction between the support and the phosphonic acid moiety leading to heterosupramolecular structures [115]. Some phosphonic acids were also considered for their flame retardant properties [116].

All these applications, that cover a very broad panel of scientific topics, point out the great interest of phosphonic acids.The next sections report the most important and efficient methods to produce phosphonic acids.

### 3. Preparation of phosphonic acids from phosphonates

#### 3.1. Hydrolysis of phosphonates with hydrochloric acid

**Figure 5:**
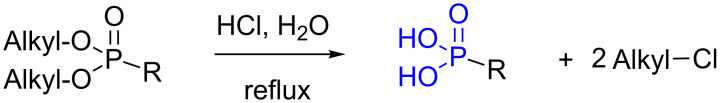
Hydrolysis of dialkyl phosphonate to phosphonic acid under acidic conditions.

The most general method to prepare phosphonic acids from phosphonates is to use concentrated HCl solution (35–37% in water; ≈12 M) at reflux for 1 to 12 h as shown in Figure 5. At the end of the reaction the excess of HCl and water can be readily eliminated by distillation and finally, the last traces of water can be removed by azeotrope distillation with toluene. Phosphonic acids, that can be very hygroscopic, can be further dried by leaving the product in a desiccator over P_2_O_5_ but, usually, they do not require further purification. When further purification is required, purification can be achieved by recrystallization in polar solvents (e.g., acetonitrile [106], methanol/acetone [117], water [55]), or in concentrated HCl [118] (acidic media reduces the water solubility of phosphonic acids). When recrystallized in aqueous solution, the phosphonic acid can co-crystallize with water due to the presence of hydrogen bonds between the phosphonic acid moiety and H_2_O [119].

**Figure 6:**
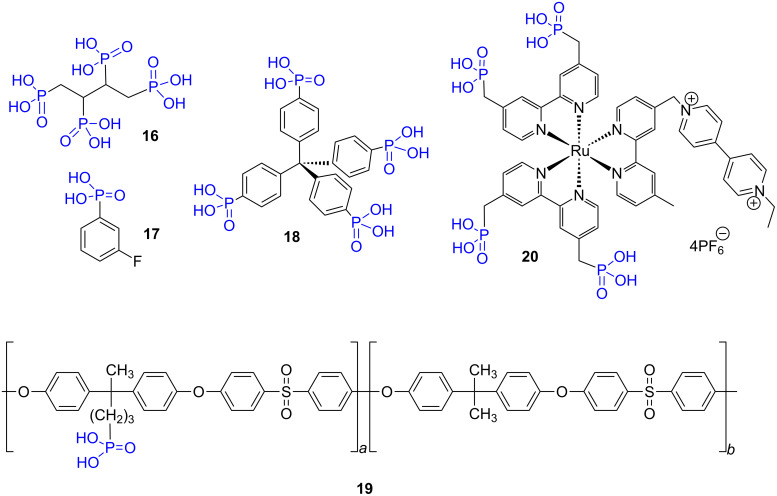
Examples of phosphonic acids prepared by hydrolysis of dialkylphosphonate with HCl 35% at reflux (**16**–**19**) or 20% HCl solution (**20**).

As depicted on Figure 6, the selected phosphonic acids prepared by the hydrolysis of phosphonate with concentrated HCl water solution present only few other functional groups. This methods was applied to prepare the alkylphosphonic acid **16** [120], arylphosphonic acid **17** [121] or **18** or block co-polymers possessing pendant arms functionalized with phosphonic acids (compound **19** [122]). The hydrolysis with 20% HCl solution (≈6 M) was also occasionally applied to coordination complexes bearing a phosphonate function as exemplified with compound **20** [123].

**Figure 7:**
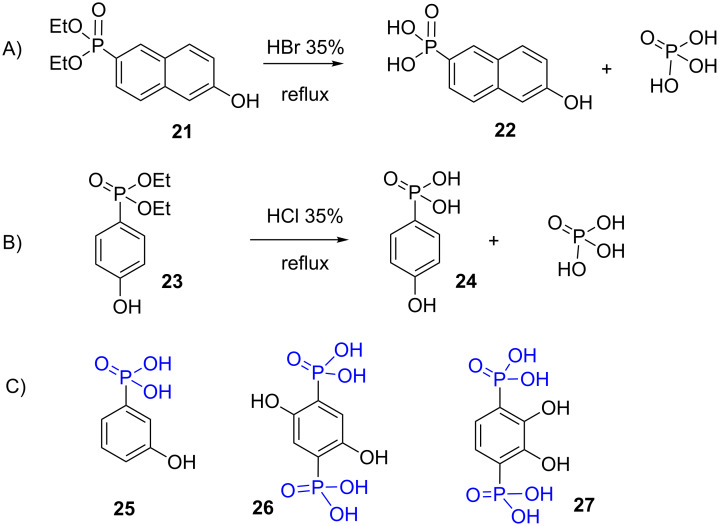
A) and B) Observation of P–C bond breaking during the hydrolysis of phosphonate with concentrated HX water solutions; C) examples of compounds prepared by hydrolysis of dialkyl phosphonate in presence of a 35% HCl–water solution without the observation of a P–C bond cleavage.

HBr was used more occasionally to hydrolyze phosphonates [124–126]. Regarding the nature of the alkyl chains of dialkyl phosphonates, it must be noted that most of the time these chains are methyl, ethyl, isopropyl or, occasionally, *n*-butyl [127]. This observation is likely explained by the synthetic methods employed to prepare phosphonates that frequently made use of the Arbuzov reaction [128] involving the commercially available trimethyl, triethyl or triisopropyl phosphite [129]. To check the achievement of phosphonate hydrolysis, phosphorus NMR is a method of choice and can also eventually detect side reactions. Indeed, with some substrates it was found that the P–C bond can be cleaved when treated with concentrated HCl as first reported by Redmore et al. [130]. For instance, hydroxynaphthylphosphonate **21** when treated with HBr induced partial rupture of the P–C bond to yield phosphoric acid which is difficult to remove from the expected phosphonic acid **22** (Figure 7A) [131]. The same side reaction was observed in the course of the acidic hydrolysis of 4-hydroxybenzenephosphonate **23** into phosphonic acid **24** (Figure 7B). However, the regioisomer diethyl 3-hydroxyphenylphosphonate can be converted to phosphonic acid **25** with HCl 35% at reflux without any P–C bond cleavage (unpublished result). The diphosphonic acids **26** and **27** were also prepared by hydrolysis with HCl 35% without P–C bond cleavage. These results indicated that the mesomeric effect induced by the phenol function likely explained the difference of stability of compounds **23** and diethyl 3-hydroxyphenylphosphonate in refluxing HCl 35% solution. Of note, if classical heating at 100 °C is usually employed some reaction under microwave activation was reported to reduce the reaction time up to only 7 minutes [132].

**Figure 8:**
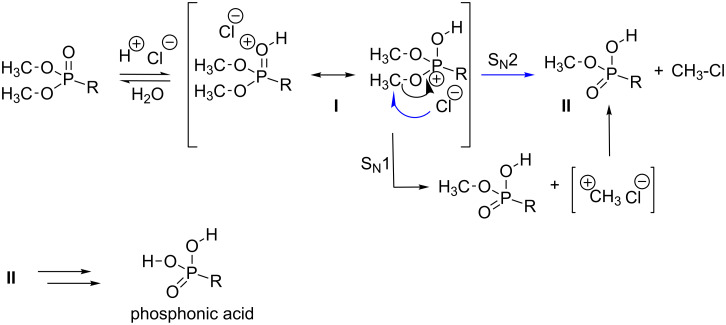
Mechanism of the hydrolysis of dialkyl phosphonate with HCl in water.

For the mechanism of hydrolysis of phosphonate by hydrochloric acid, some works dedicated to assess the hydrolysis of trimethyl phosphate are instructive and can likely be extrapolated to phosphonate. When trimethyl phosphate (TMP) is placed in 4 N HCl solution, infrared analysis showed a shift of the P=O band from 1273 cm^−1^ to 1236 cm^−1^ [133]. When the HCl concentration was further increased to 12 N, this band disappeared. The titration of trimethyl phosphate with HCl was also monitored by ^31^P NMR and the results are consistent with a protonation at the oxygen atom that is doubly bonded to the phosphorus atom leading to a phosphorus centered cation (the protonated trimethyl phosphate was characterized by a p*K*_a_ of −3.6). These results, extrapolated to phosphonate, suggest that the protonation of the phosphonate would occur on the oxygen atom which is doubly bonded to the phosphorus atom. This protonation likely yields the intermediate **I** (Figure 8) which exists as two mesomeric forms. Then, two competitive mechanisms can occur. Intermediate **I** can lose a carbocation according to a S_N_1 mechanism whereas the second way could consist in a nucleophilic substitution (S_N_2) involving chloride ions as nucleophilic species to produce the intermediate **II**. Then, a repetition of this mechanism yields phosphonic acid. The preponderant route is likely governed by the stability of the carbocation and the steric hindrance around the electrophilic carbon atom of intermediate **I** (Figure 8).

**Figure 9:**
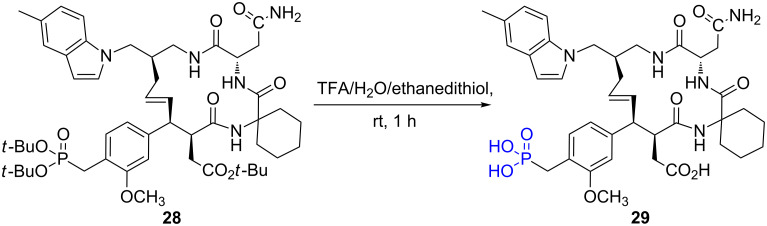
Hydrolysis of bis-*tert*-butyl phosphonate **28** into phosphonic acid **29** [137].

Di-*tert-*butyl phosphonate belongs to the precursors of phosphonic acid that requires milder acidic conditions to be converted into phosphonic acid. The mechanism likely occurs following the S_N_1 pathway as reported above. This type of di-*tert*-butyl phosphonate was used for the synthesis of phosphonic acid analogues of peptides like protein-tyrosine kynases (PTKs) [134] or to prepare gadolinium complexes used as magnetic resonance imaging (MRI) contrast agents [135–136]. The introduction of the di-*tert*-butyl phosphonate was achieved by the reaction between di-*tert*-butyl phosphite and either benzyl halide [137] (Michaelis–Becker reaction) or aldehyde [134] (Pudovik reaction) or using tris-*tert*-butyl phosphite (Abramov reaction) [135]. The elimination of the *t*-Bu moieties can be efficiently achieved in presence of trifluoroacetic acid (TFA). As exemplified in Figure 9, the use of TFA induces the formation of the phosphonic acid **29** concomitantly with the formation of carboxylic acid from the *tert*-butyl ester. The final compounds were purified by removing the excess of TFA (72.4 °C at 1 bar) [138], using HPLC [137] or by precipitation in diethyl ether [136].

**Figure 10:**
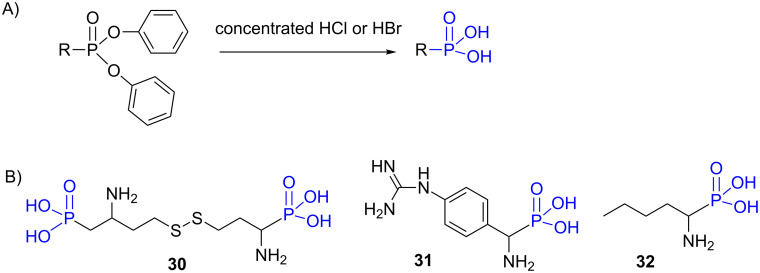
A) Hydrolysis of diphenyl phosphonate into phosphonic acid in acidic media. B) Examples of phosphonic acids prepared by this method.

All the precedent examples illustrate the hydrolysis of dialkyl phosphonate in phosphonic acid under acidic conditions. Similar protocols (concentrated HCl or HBr) can be used to prepare phosphonic acid from diphenyl phosphonate (Figure 10A). The use of a mixture of HBr in acetic acid was also reported [139–140]. It must be noted that the reaction work-up is usually slightly different since the treatment with propylene oxide that acts as an acid scavenger, can be applied during the purification step. The preparation of phosphonic acid from diphenyl phosphonate was reported to prepare phosphohomocysteine **30** [141], the arginine mimetic **31** that was developed as a potent metallo-aminopeptidase inhibitor [142] or the aminophosphonic acid **32** [143] (Figure 10B).

**Figure 11:**
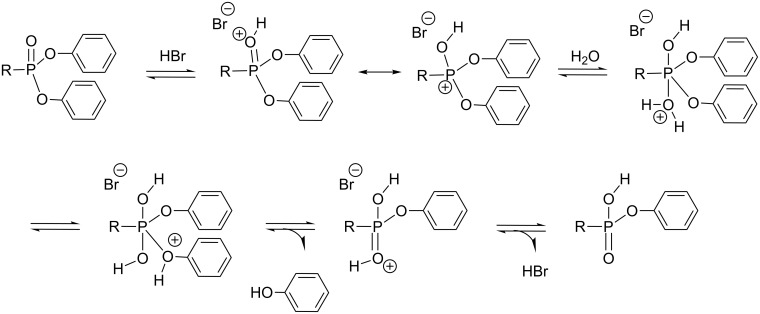
Suggested mechanism occurring for the first step of the hydrolysis of diphenyl phosphonate into phosphonic acid.

The mechanism of hydrolysis of diphenyl phosphonate in acidic conditions is likely different to the one occurring with dialkyl phosphonates (Figure 8). For the hydrolysis of diphenyl phosphonate it is likely that after protonation of the phosphonate, water acts as a nucleophile and subsequently phenol is eliminated (Figure 11). The repetition of this sequence produces phosphonic acid.

Of note, the treatment of phosphonate with nucleophilic basic reagents like NaOH [144], LiOH [145] or NaHCO_3_ [146] is not suitable to produce phosphonic acid. Indeed, these conditions yield phosphonic acid mono-esters. This monohydrolysis takes place with dialkyl phosphonate or diphenyl phosphonate [146]. For dimethyl phosphonate, the monohydrolysis can be also achieved using NaI as nucleophile in acetone [147] or butanone as solvent [148].

#### 3.2. Catalytic hydrogenolysis

**Figure 12:**
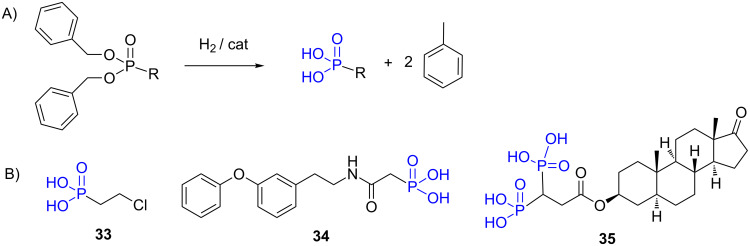
A) Hydrogenolysis of dibenzyl phosphonate to phosphonic acid. B) Compounds **33**, **34** and **35** were prepared by hydrogenolysis on Pd/C from dibenzyl phosphonates.

Dibenzyl phosphonates are readily synthesized using dibenzyl or tribenzyl phosphite. This type of phosphonate offers an alternative to the use of acidic conditions to prepare phosphonic acid since the benzyl moieties can be removed by hydrogenolysis (Figure 12). Palladium on charcoal, which is the most used method to prepare phosphonic acid from dibenzyl phosphonate [149], was the catalyst used to prepare the phosphonic acids **33** [150], **34** [151] and **35** [152] (Figure 12). Of note, mono-hydrolyzed phosphonate can be obtained when a monoalkyl-monobenzyl phosphonate is hydrogenolyzed [150,153].

**Figure 13:**
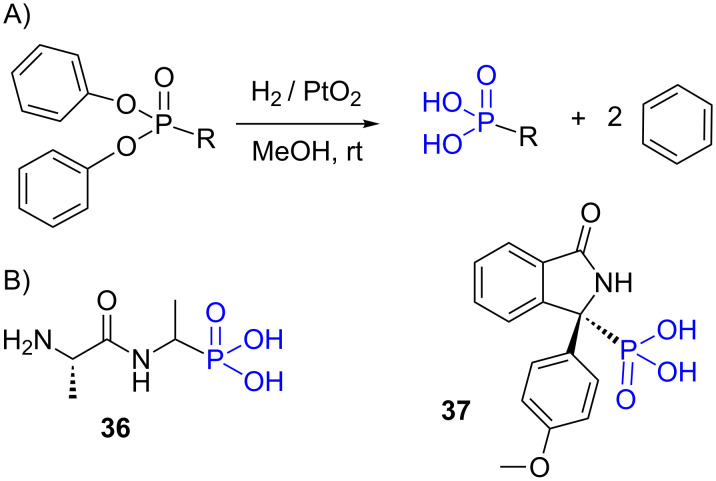
A) Preparation of phosphonic acid from diphenyl phosphonate with the Adam’s catalyst. B) Compounds **36** and **37** were prepared with this method.

The synthesis of phosphonic acid from diphenyl phosphonates can be prepared under acidic conditions (see above) but can be also achieved in presence of Adam’s catalyst (PtO_2_) and hydrogen as exemplified by the preparation of the compounds **36** [154] and **37** [155] (Figure 13).

It is also worth noticing that phosphonic acid can be prepared by the hydrogenolysis of diallyl phosphonates. For this purpose the use of the Wilkinson catalyst (ClRh(PPh_3_)_3_) was reported [156].

#### 3.3. McKenna’s method – use of bromotrimethylsilane

**Figure 14:**
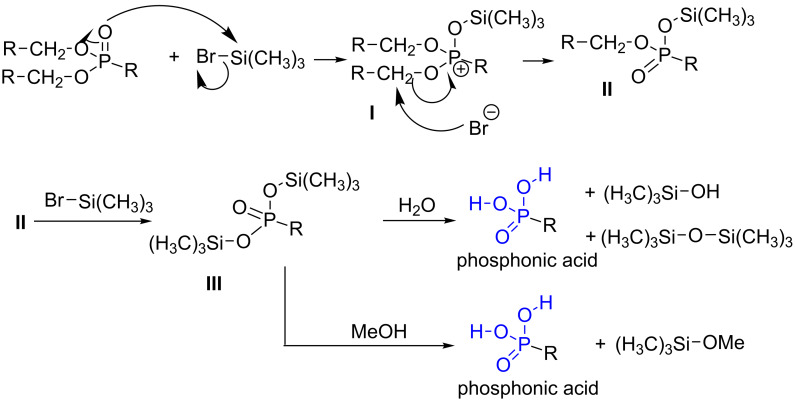
Suggested mechanism for the preparation of phosphonic acid from dialkyl phosphonate using bromotrimethylsilane.

As reported above, the hydrolysis in acidic media requires harsh conditions (most of the time concentrated HCl solution in water at reflux) that finally limit its application when the molecules possess sensitive functional groups. The need of a milder method to prepare more functionalized phosphonic acid derivatives was therefore required. In some aspect the selection of benzyl or *tert*-butyl phosphonate can be suitable since specific mild conditions can be applied to prepare phosphonic acids as discussed above. Nevertheless, as the simplest way to prepare phosphonate involves trialkyl phosphite, the subsequent dealkylation of dialkyl phosphonate under mild conditions was of a great importance. The group of C. E. McKenna reported in 1977 the use of bromotrimethylsilane as a reagent that permitted an efficient transesterification of dialkyl phosphonate to bis-(trimethylsilyl) phosphonate. It is worth noticing that these silylated phosphonates produced quantitatively phosphonic acid derivatives after water or alcohol (methanol, ethanol) treatment [157]. In this initial work, NMR data indicated that silylation of dimethyl or diethyl phosphonate with bromotrimethylsilane was almost quantitative. Other studies indicated that this reaction can be also applied to diisopropyl phosphonate, or di-*tert*-butyl phosphonate leading to the conclusion that all dialkyl phosphonates can be dealkylated by the transesterification followed by a methanolysis or hydrolysis subsequent step. As suggested in the initial works of McKenna, the mechanism occurs by an oxophilic substitution on the silicon atom whereas bromide acts as a leaving group to produce the intermediate **I** (Figure 14). This intermediate is then dealkylated following a similar path than that occurring with the Arbuzov reaction to produce the intermediate **II** [158]. The repetition of this mechanism produced de disilylated intermediate **III**. The hydrolysis of this intermediate **III** produced phosphonic acid, trimethylsilanol and hexamethyldisiloxane that are two volatile side products. The methanolysis of the intermediate **III** is even a better choice to transform **III** into phosphonic acid since the methoxytrimethylsilane is also volatile and methanol used in excess is also more volatile than water. It must be noted that an experimental proof (using ^17^O- and ^18^O-enriched diethyl phenylphosphonate) has reported that the terminal oxygen doubly bonded to the phosphorus atom was indeed the nucleophilic atom that attacks the silicon atom [159].

**Figure 15:**
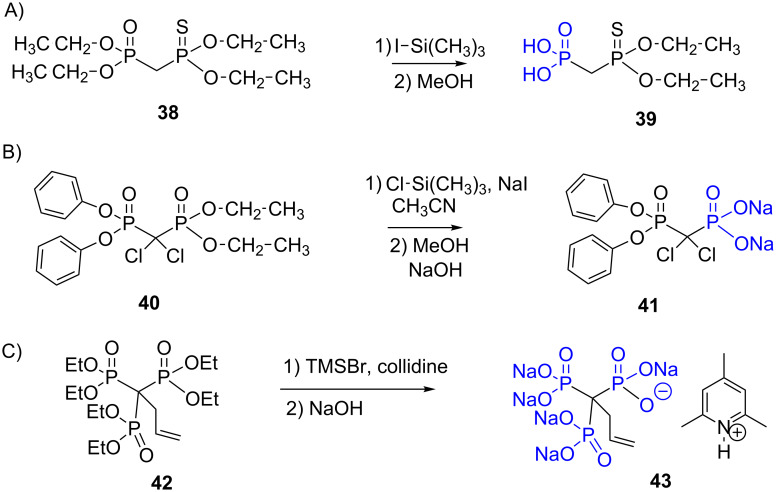
A) Reaction of the phosphonate-thiophosphonate **37** with iodotrimethylsilane followed by methanolysis. B) Illustration of the selectivity of BrSiMe_3_towards dialkyl phosphonate versus diaryl phosphonate. C) Use of bromotrimethylsilane to prepare the 3-butenyltrisphosphonic acid pentasodium 2,4,6-trimethylpyridinium salt **42**.

Regarding the mechanism of this reaction, the oxophilic silylation involving the oxygen atom which is doubly bonded to the phosphorus atom, thus producing the intermediate **I**, is also supported by the works of Bartlett et al. [160] (Figure 15A) that show that the treatment of the phosphonate-thiophosphonate **38** with iodotrimethylsilane produced, after methanolysis, only the phosphonic acid **39**. Of note, the thiophosphonate functional group is not hydrolysed. The need of the nucleophilic halide anion is another feature of this reaction. Indeed the treatment of TMSOTf on a phosphonate did not induce the dealkylation likely due to the absence of nucleophilic species [161].

It is worth noticing that the nucleophilic attack of bromide only occurs on alkyl chains as exemplified by a work of Pohjala et al. [162] (Figure 15B) The authors showed that the diphosphonate **40** featuring both phenyl and alkyl substituents treated with chlorotrimethylsilane and sodium iodide in CH_3_CN yielded, after methanolysis and treatment with NaOH, compound **41** resulting from the hydrolysis of the diethyl phosphonate moiety. Finally, it must be noted that BrSiMe_3_ selectively induced the silylation of phosphonate without affecting ketone, amide, halogenoalkane or alkyne [163]. The preparation of phosphonic acid from phosphonate with bromotrimethylsilane was also applied to methylene-tris-phosphonic acids [164], and to substituted hexaethyl 1,1,1-tris-phosphonate as illustrated with the reactivity of compound **42**. This reaction occurred in presence of collidine to prepare, after treatment with sodium hydroxide, the collidinium salt **43** [165] (Figure 15C).

**Figure 16:**
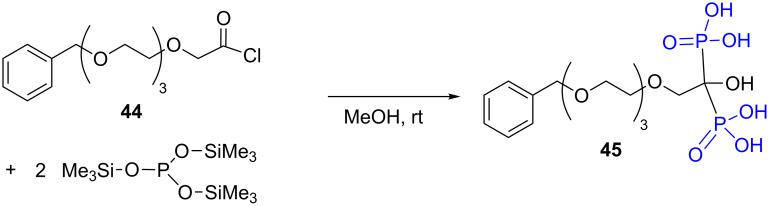
Synthesis of hydroxymethylenebisphosphonic acid by reaction of tris(trimethylsilyl) phosphite with acyl chloride.

The mechanism of dealkylation with bromotrimethylsilane, that produces a bis-silylated phosphonate intermediate, thus incited to use tris(trimethylsilyl) phosphite as nucleophilic phosphorus species to produce after methanolysis phosphonic acid [166] or hydroxymethylene bis-phosphonic acid as illustrated in Figure 16 with the synthesis of the hydroxymethylenebisphosphonic acid **45** [167].

**Figure 17:**
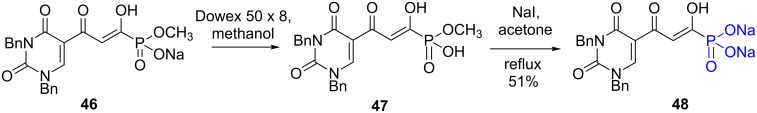
Synthesis of the phosphonic acid disodium salt **48** by reaction of mono-hydrolysed phosphonate **47** with NaI.

The trans-esterification of phosphonate with bromotrimethylsilane to produce silylated phosphonate requires at least two equivalents of BrSiMe_3_ per phosphonate function. Usually an excess is engaged in the reaction to guarantee a full conversion of dialkyl phosphonate into the disilylated phosphonate. The same reaction is also observed with the use of iodotrimethylsilane (Me_3_SiI) [168]. However, with chlorotrimethylsilane the reaction is not really efficient as indicated in the initial work of McKenna since only a partial conversion was observed after several days of reaction. Nevertheless, the use of a mixture of chlorotrimethylsilane with NaI in acetonitrile can be used to achieve the silylation of dialkyl phosphonates that can be then transformed in phosphonic acids after hydrolysis or methanolysis. Following these experimental conditions, the efficacy is likely explained by the in situ generation of iodotrimethylsilane that result from a halogen exchange reaction [169]. This method (ClSiMe_3_ + NaI in acetonitrile) is currently less employed likely due to the need to remove NaI from the final phosphonic acid after the step of hydrolysis or methanolysis. It is worth noticing that when NaI, LiBr or KI was used alone in anhydrous solvents (acetone, MeCN or butanone) under heating (80–100 °C) a selective monodeprotection of the dialkyl phosphonates (alkyl = methyl or ethyl) was observed. The sodium or lithium salts being formed in high yields (87–97% yields) [170]. This procedure was applied to prepare a molecular receptor of lysine residue [171]. Following this procedure, the monodealkylation can be explained by the formation of a sodium salt that due to electronic factors prevent the second dealkylation. However, the protonation of this anionic compound **46** by ion exchange procedure yields **47** that can then be fully dealkylated with NaI in acetone to produce the phosphonic acid disodium salt **48** [172] (Figure 17).

**Figure 18:**
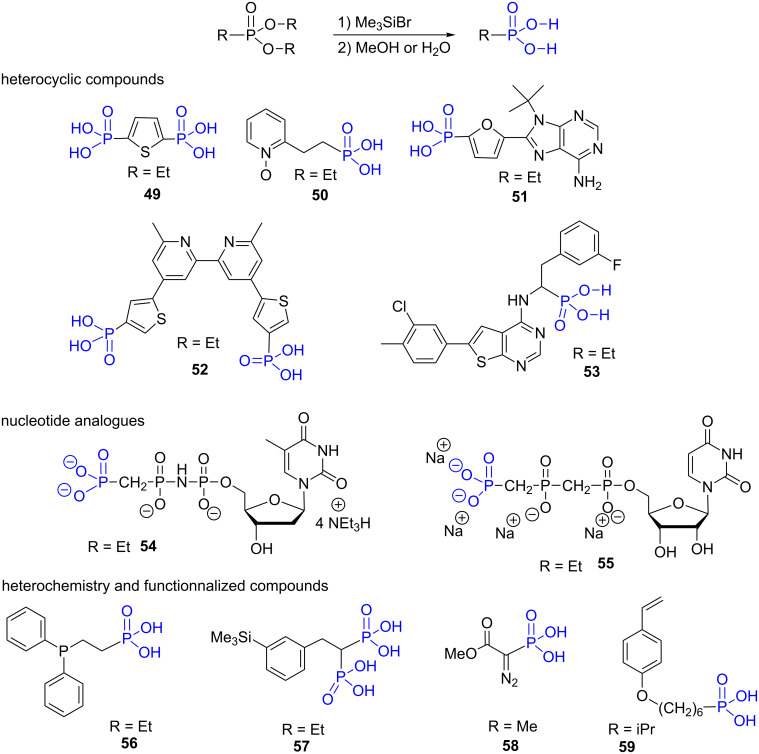
Phosphonic acid synthesized by the sequence 1) bromotrimethylsilane 2) methanolysis or hydrolysis. The notation R indicates the nature of the alkyl chains present on the phosphonate precursors of these phosphonic acids.

Bromotrimethylsilane is nowadays the gold standard reagent to produce phosphonic acid from dialkyl phosphonate under mild conditions (usually at room temperature). This reaction can be achieved in a non-protic solvent including CH_2_Cl_2_ [173], acetonitrile [174], chloroform [175], DMF [176], pyridine [177] or collidine [178]. The use of BrSiMe_3_ simultaneously as reagent and solvent was also reported [120]. The direct production of sodium salts of phosphonic acid is readily achieved by adding 2 N NaOH/water solution to the silylated phosphonate [178]. This two-step sequence: 1. bromotrimethylsilane; 2. methanolysis or hydrolysis was used for the synthesis of numerus phosphonic acids including heterocyclic compounds that are too sensitive to be prepared by the transformation of phosphonates under acidic conditions. As an illustration, the thiophene diphosphonic acid **49** [173], the pyridine oxide **50** [179], the furane phosphonic acid **51** [174], the bipyridine bis thiophene phosphonic acid **52** [180] or the α-aminophosphonic acid **53** that was assessed as inhibitor of the human farnesyl pyrophosphate synthase (hFPPS) [181] were prepared from their corresponding diethylphosphonates (Figure 18). Nucleotide analogues were also prepared by this methodology as exemplified by the compound **54** [176] or **55** [182]. For these last two examples, the bromotrimethylsilane induced the silylation of diethyl phosphonate but also the phosphoramidate and the phosphinate functional groups. Other phosphonic acids possessing different functionalities including phosphine **56** [183], trimethylsilyl **57** [184], diazo **58** [185] or styrene **59** [186] moieties were also prepared efficiently using bromotrimethylsilane followed by methanolysis.

**Figure 19:**
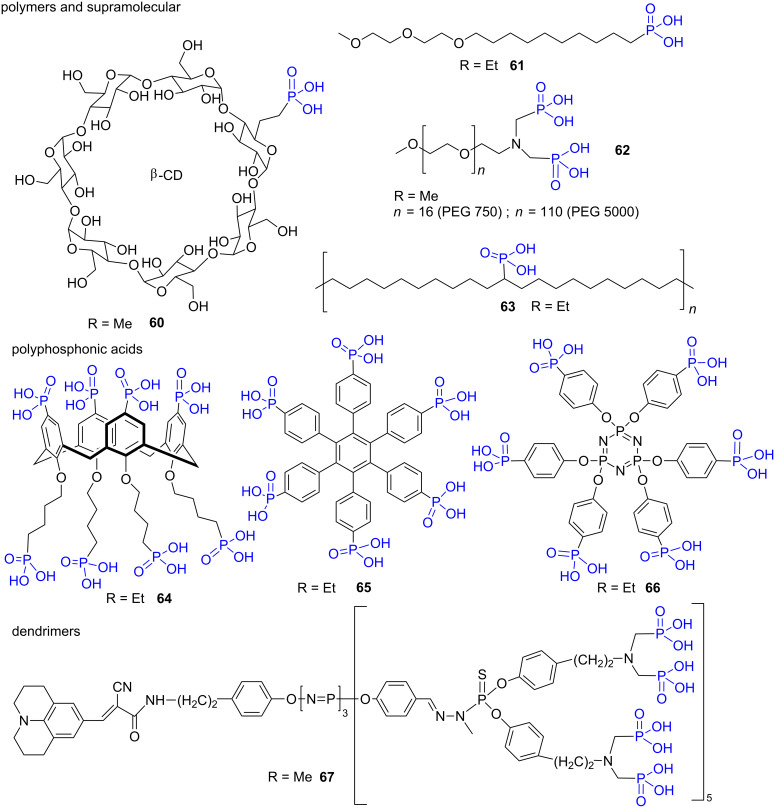
Polyphosphonic acids and macromolecular compounds prepared by the hydrolysis of dialkyl phosphonate following the McKenna’s method. The notation R indicates the nature of the alkyl chains present on the phosphonate precursors of these phosphonic acids.

This two-step methodology (1. BrSiMe_3_; 2. MeOH or H_2_O) was also applied to prepare macromolecules functionalized with phosphonic acid functional groups. For instance, a cyclodextrine derivative (compound **60** [177], Figure 19), amphiphilic compounds that were used for self-assembled monolayers (e.g., compound **61** [187]), polymers (PEG derivative **62** [188] or functionalized polyethylene **63** [189]) were reported. The McKenna’s procedure was also applied to prepare polyphosphonic acids as exemplified by the compounds **64** [120], **65** [190], **66** [191] and the dendrimers **67** [192].

**Figure 20:**
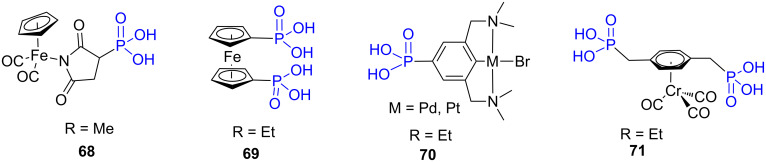
Examples of organometallic complexes functionalized with phosphonic acids that were prepared by the hydrolysis of dialkyl phosphonate according to the McKenna’s method. The notation R indicates the nature of the alkyl chains present on the phosphonate precursors of these phosphonic acids.

The use of BrSiMe_3_ was also applied to diverse organometallic compounds as exemplified in Figure 20. This procedure was applicable to compounds featuring CpFe(CO)_2 _**68** [193] or ferrocenyl **69** [194–195] moieties but also to palladium or platinium pincer complexes **70** [196] or arene–chrome carbonyl derivatives **71** [197]. It must be noted that when the organometallic complex possessed halogen–metal moiety, then Me_3_SiBr can induce halogen exchange [198].

**Figure 21:**
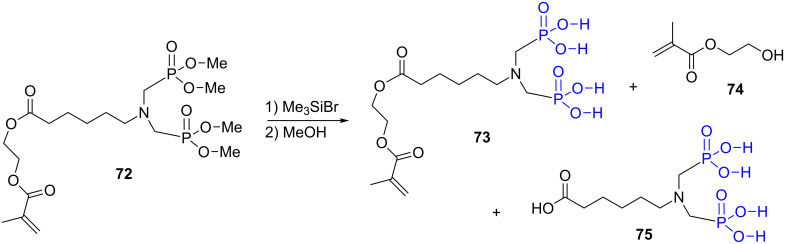
Side reaction observed during the hydrolysis of methacrylate monomer functionalized with phosphonic acid.

The use of bromotrimethylsilane to prepare phosphonic acid from dialkyl phosphonate is rarely associated with undesired side reactions. Nevertheless, G. David et al. have reported in a study dedicated to the synthesis of methacrylate monomers functionalized with phosphonic acids the occurrence of side compounds [199]. The treatment of the bis-phosphonate **72** with TMS-Br followed by methanolysis, induces a cleavage of the carboxylic esters simultaneously with the formation of the phosphonic acid **75** (Figure 21). The authors have shown, using model compounds, that the hydrolysis is triggered by the acidity of the phosphonic acid. Interestingly, the addition of aqueous ammonia during methanolysis of the disilylated phosphonate produced the expected phosphonic acid **73** without any cleavage of the carboxylic ester function.

**Figure 22:**
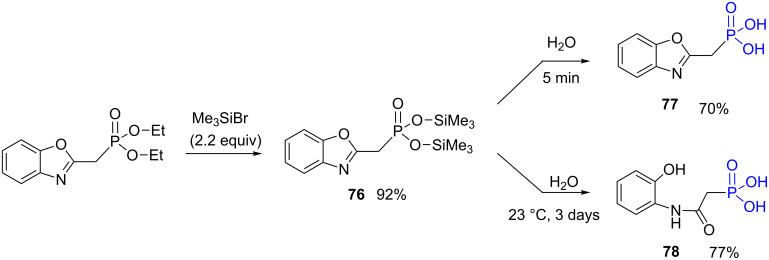
Influence of the reaction time during the hydrolysis of compound **76**.

A second example reported by Pailloux et al. shows that depending on the reaction time for the hydrolysis of the silylated phosphonate **76** (Figure 22) either the expected phosphonic acid **77** or the phosphonic acid **78** resulting from the opening of the benzoxazole fragment (after 3 days in contact with water), were produced [200]. This side reaction is likely explained by the sensitivity of the product to acidic condition coming from the acidity of the phosphonic acid function. During the hydrolysis the precipitation of the phosphonic acid **77** occurred; a rapid filtration (5 minutes) led to isolate the phosphonic acid **77** in 70% yield. However, a prolonged contact time with water (3 days) yields the phosphonic acid **78** that features a benzoxazole ring opening. It can be concluded form these studies (Figure 21 and Figure 22) that the side reactions is not due to bromotrimethylsilane but to the sensitivity of the product to acidic conditions that are induced by the acidity of the phosphonic acid functional group.

#### 3.4. Dealkylation with boron reagents

**Figure 23:**
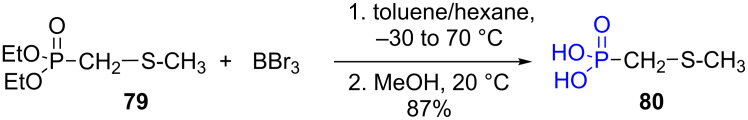
Dealkylation of dialkyl phosphonates with boron tribromide.

Dealkylation of dialkyl phosphonates could also be performed using C_6_H_11_BCl_2_ [201] or BBr_3_ [202] under mild and efficient conditions as reported by Mortier et al. The reaction can be applied to dialkyl phosphonates (R = Me, Et, iPr, *t*-Bu) and proceed first at −30 °C and then at 70 °C in an aprotic solvent (toluene) for 6 hours (Figure 23). The authors reported the formation of a primary adduct R_3_B···O=PR(OR')_2_ which was subsequently decomposed as borophosphonates oligomers through a bis-bromodealkylation [203]. The phosphonic acids were finally obtained after methanolysis with quantitative conversion and yields up to 95%_._ Interestingly, this procedure is selective to P–O dealkylation and compatible with the presence of other groups such as allyl, ketone, primary alcohol, phthalimide, ester or thioether as illustrated with the transformation of the phosphonate **79** to the phosphonic acid **80** (Figure 23). The best results were obtained when 0.9 equivalents of BBr_3_ per phosphonate function was used.

#### 3.5. Other conditions for dealkylation of phosphonate

**Figure 24:**
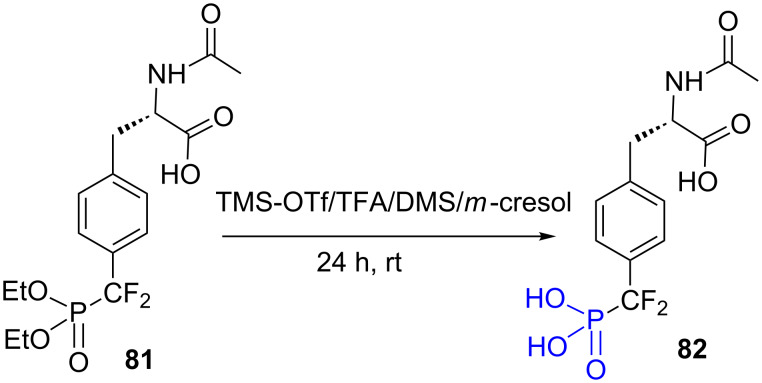
Dealkylation of diethylphosphonate **81** with TMS-OTf.

The dealkylation of diethyl phosphonate groups present in amino acids or peptides requires mild conditions to avoid the reactivity with the other functional groups. If Me_3_SiBr can be used to dealkylate peptides functionalized with diethyl phosphonate groups [204], other mild conditions, developed in the context of peptide chemistry, were reported. It is based on the use of trimethylsilyl trifluoromethanesulfonate (TMS-OTf) as silylating agent, trifluoroacetic acid (TFA) as acidic reagent, and dimethylsulfide (DMS) and *m*-cresol to avoid side reactions (from S_N_1 or S_N_2 mechanism) [204] that can involve the functional groups present in the amino acid or peptide. These conditions were applied to prepare the compound **82** from **81** [205–206] (Figure 24).

### 4. From dichlorophosphine (R–PCl_2_) or dichlorophosphine oxide (R–P=OCl_2_)

**Figure 25:**
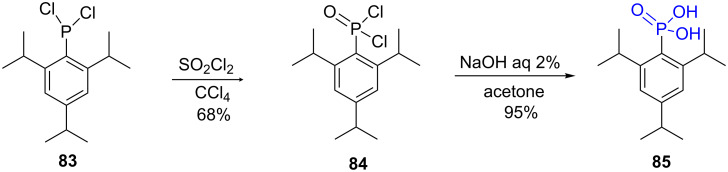
Synthesis of substituted phenylphosphonic acid **85** from the phenyldichlorophosphine **83**.

Aryl- and to a lesser extent alkylphosphonic acids have been prepared by the hydrolysis under mild conditions of aryldichlorophosphine or aryldichlorophosphine oxide. The hydrolysis is often performed under basic conditions using aqueous sodium hydroxide (5% NaOH); the phosphonic acid being lately isolated by acidification with hydrochloric acid. For example, the sterically hindered phenylphosphonic acid **85** was prepared in a two-step procedure starting from the dichlorophenylphosphine **83**. Ph–PCl_2_ was first oxidized into the phenylphosphine oxide **84** with sulfuryl chloride in CCl_4_ and then hydrolysis occurred with aqueous NaOH to afford the corresponding arylphosphonic acid **85** in good yield (65% yield, 2 steps) as shown in Figure 25 [207].

**Figure 26:**
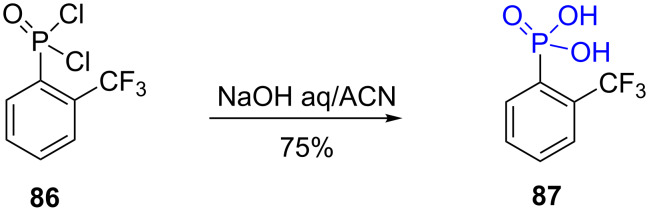
Hydrolysis of substituted phenyldichlorophosphine oxide **86** under basic conditions.

More recently, the synthesis of *o*-trifluoromethylbenzenephosphonic acid (**87**) was prepared by hydrolysis of the phenyldichlorophosphine oxide **86** with NaOH in acetonitrile (75% yield, Figure 26). The hydrolysis in acidic conditions (10% HCl) was also reported and produced the phosphonic acid with similar yield (75–85%). However, hydrolysis under acidic conditions required an extended reaction time [208].

### 5. Phosphonic acid from phosphonodiamide RP=O(NR_2_)_2_

**Figure 27:**
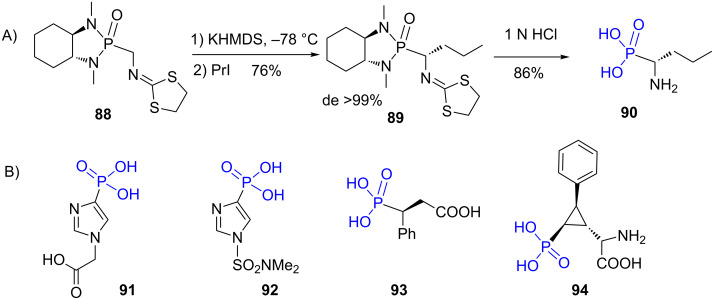
A) Illustration of the synthesis of chiral phosphonic acids from phosphonodiamides. B) Examples of phosphonic acid prepared by acidic hydrolysis of phosphonodiamides.

Phosphonodiamides are commonly used as precursors of phosphonic acids especially for the synthesis of aminophosphonic acids (α [209–210], β [211] or γ [212]) and also for the synthesis of nucleoside analogues [213]. The use of these intermediates is likely explained by the possibility to use chiral diamine that can act as a chiral auxiliary to control the chirality of the carbon atom in α-position of the phosphorus atom as exemplified by the alkylation of the phosphonodiamide **88** (Figure 27). Furthermore, phosphonodiamide can be purified by different methods including chromatography and their transformation to phosphonic acid is easily achieved by hydrolysis under acidic conditions as illustrated by the reaction of compound **89** that produced the α-amino-phosphonic acid **90** after acidic hydrolysis [210] (Figure 27). Similar acidic hydrolysis was used to produce the imidazolyl phosphonic acid **91** [214], **92** [215], the chiral phosphonic-carboxylic acid **93** [216] or the cyclopropylphosphonic acid **94** that was assessed as inhibitor of the glutamate metabotropic receptors [217].

**Figure 28:**
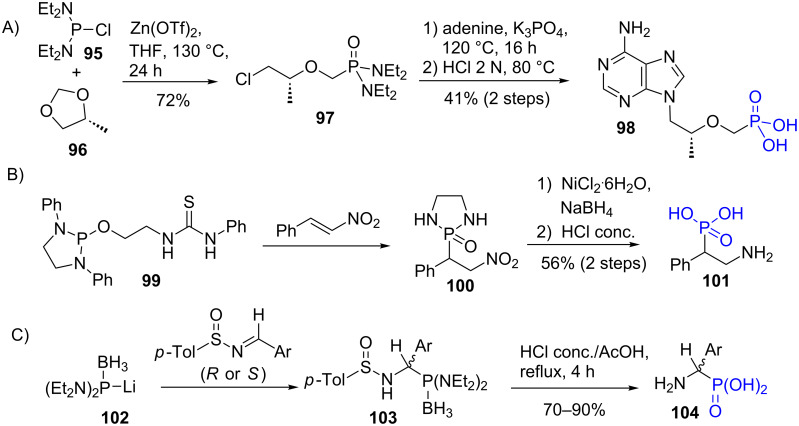
A) Illustration of the synthesis of the phosphonic acid **98** from phosphonodiamide **97**. B) Use of cyclic phosphonodiamide **100** to prepare phosphonic acid. C) Illustration of the use of lithiated bis(diethylamino)phosphine borane complex **102** to prepare phosphonic acid.

The synthesis of phosphonic acid via phosphonodiamide intermediates started by the use of the nucleophilic bis(dialkylamino)chlorophosphine. This possibility is illustrated by the recent works reporting the reaction of bis(diethylamino)chlorophosphine (**95**) with the acetal **96** in the presence of a Lewis acid to yield the phosphonodiamide **97**. Then the nucleophilic addition of adenine and the hydrolysis of the phosphonodiamide function in phosphonic acid produce compound **98** [213] (Figure 28A). A second example corresponds to the nucleophilic addition of *N*-heterocyclic phosphine **99** to a nitroalkene (phospho-Michael reaction) as shown in Figure 28B. The thiourea unit in **99** plays a crucial role by assuming intramolecular interaction with the nitro function that finally led to the formation of the phosphonodiamidate **100**. The reduction of the nitro function and the hydrolysis under acidic conditions of the phosphonodiamide produced the phosphonic acid **101** [211]. As a last example, lithiated diaminophosphine borane **102** was added to imine to produce the bis(diethylamino)phosphone borane **103** (Figure 28C). Then acidic hydrolysis conditions and likely air oxidation produced the phosphonic acid **104** [209].

**Figure 29:**
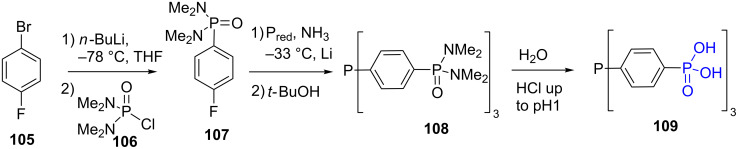
Synthesis of tris(phosphonophenyl)phosphine **109**.

Phosphonodiamide, which is more robust than phosphonate towards some nucleophilic species like a phosphide anion (R_2_P-Li), was used to prepare triarylphosphine functionalized with three phosphonic acid groups (Figure 29) [24]. The sequence requires the preparation of the 4-fluorophenylphosphonodiamide **107** which is prepared by nucleophilic addition of an aryllithium intermediate (from **105**) on chlorophosphadiamide **106**. In the last step, the tris(phenylphosphonodiamide)phosphine **108** was hydrolysed into the tris-phosphonic acid **109** using water and HCl up to pH 1. Of note, the same reaction that engaged diethyl 4-fluorophenylphosphonate instead of the 4-fluorophenylphosphonodiamide **107** is inefficient (3% yield) due to the monohydrolysis of the phosphonate.

### 6. Direct method from phosphorous acid H_3_PO_3_

The direct methods correspond to the methods where the formation of the P–C bond yields simultaneously phosphonic acid. These reactions mainly use phosphorous acid (H_3_PO_3_) as source of phosphorus. The main difficulty with such type of reaction is the purification of the product since crystallization, precipitation and dialysis constitute the possible methods of purification. The purification by chromatography requires, due to the high polarity of phosphonic acids, reversed-phase chromatography and is therefore limited to preparative RP-HPLC [218]. Despites these difficulties, some reactions are very efficient as reported below.

#### 6.1. Moedritzer–Irani reaction

**Figure 30:**
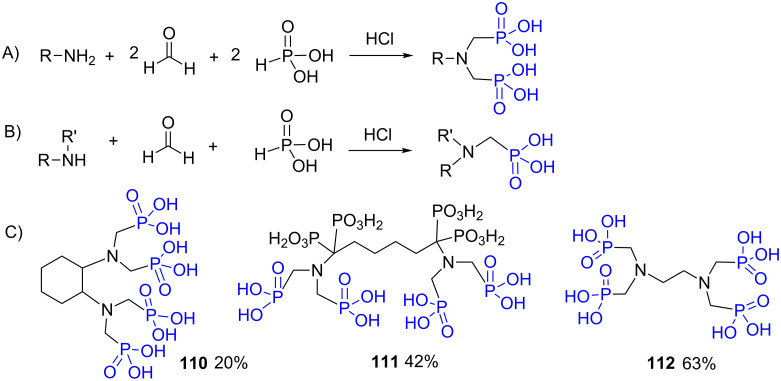
Moedritzer–Irani reaction starting from A) primary amine or B) secondary amine. C) Examples of phosphonic acids prepared by the Moedritzer–Irani reaction.

The reaction of an amine (primary or secondary amine), formaldehyde (aqueous or solid paraformaldehyde) and phosphorous acid in acidic media produces amino-bis(methylenephosphonic acid, Figure 30A,B). This reaction, is also known as the Moedritzer–Irani reaction since it was first reported by these authors in 1966 [219]. When primary amine is used, the reaction produces a bis-phosphonic acid since the secondary amine that is generated in situ is more reactive than the starting primary amine [220]. In consequence, the stoichiometry must be adapted and 2 equivalents of both formaldehyde and phosphorous acid must be used. The reaction is also applicable to secondary amine and produce mono-aminomethylene phosphonic acid (Figure 30B). The yield of this reaction is variable as illustrated in Figure 30C. The compound **110** was obtained in 20% from diaminocyclohexane [221]. The compound **111** was prepared from a bis(α-aminomethylene diphosphonic acid) and was isolated in 42% yield. This compound was assessed as a scale inhibitor [222]. As a last example, the compound **112** was prepared from diaminoethane using microwave heating. The final product was isolated in 63% yield [223].

**Figure 31:**
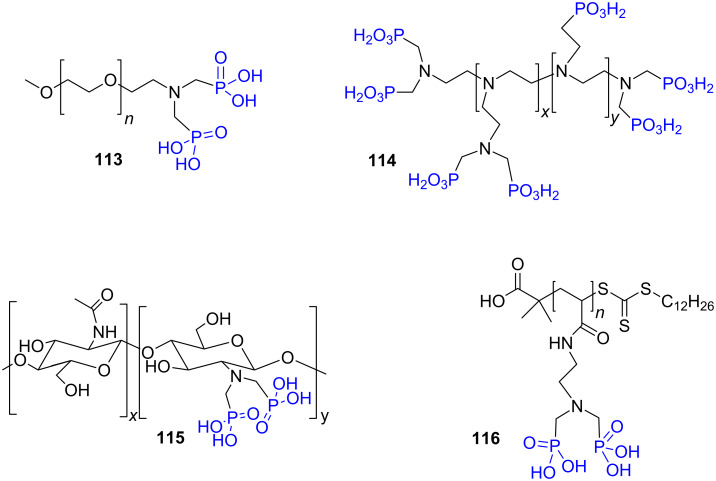
Phosphonic acid-functionalized polymers prepared by Moedritzer–Irani reaction.

The Moedritzer–Irani reaction was also applied to introduce phosphonic acid functions on polymer or dendrimers possessing reactive amine. Accordingly, phosphonic acids were introduced on polyethyleneglycol **113** [188], polyethylene imine **114** [224] or chitosan **115** [225]. The functionalization of polyacrylamide obtained by reversible addition-fragmentation chain transfer (RAFT) polymerization was also recently reported to produce **116** (Figure 31). However, the conditions of the Moedritzer–Irani reaction induced the hydrolysis of the polymers side chains [226].

#### 6.2. Reactivity of phosphorous acid (H_3_PO_3_) with imine, nitrile and carbonyl groups

**Figure 32:**
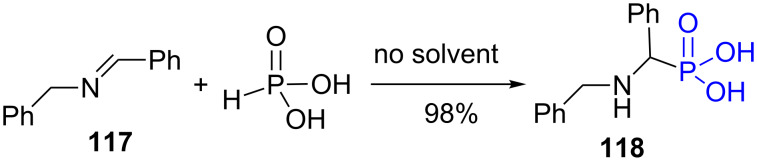
Reaction of phosphorous acid with imine in the absence of solvent.

The phosphorous acid function reacts with imine in the absence of solvent to produce α-amino-phosphonic acid as exemplified by the synthesis of the α-amino-phosphonic acid **118** from the imine **117** (Figure 32) [227]. It is worth noting that the reaction of H_3_PO_3_ with some enamine (e.g., 1-morpholinocyclohexene) gives a reduction of the enamine to amine. This is likely why this reaction was seldomly used.

**Figure 33:**
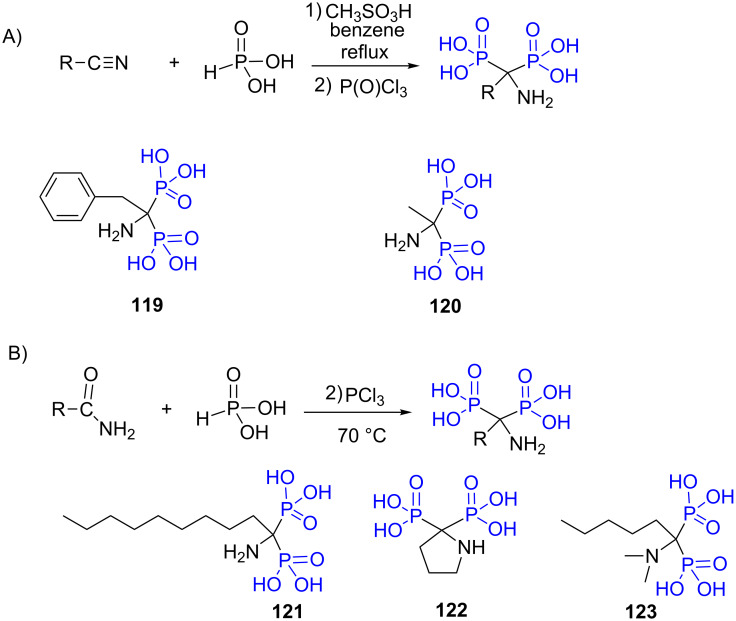
A) Reaction of phosphorous acid with nitrile and examples of aminomethylene bis-phosphonic acids. B) Reaction of phosphorous acid with amide and examples of compounds prepared by this reaction.

The reaction of phosphorous acid on nitrile in presence of methanesulfonic acid followed by the addition of POCl_3_ or PCl_3_ [222] is a method that produces in one step the aminomethylene bis-phosphonic acid [228] (Figure 33A). This methodology was applied to prepare the compound **119** [229] that was assessed as HIV-1 integrase inhibitor. Compound **120** [230] was prepared simply by the refluxing acetonitrile with phosphorous acid at 130 °C for 12 hours. **120** was a member of a series of compounds developed as a potential inhibitor of the farnesyl pyrophosphate synthase. The reaction also occurs with amide (Figure 33B) as exemplified for the synthesis of compounds **121** [231], **122** [232] or **123** [231].

**Figure 34:**
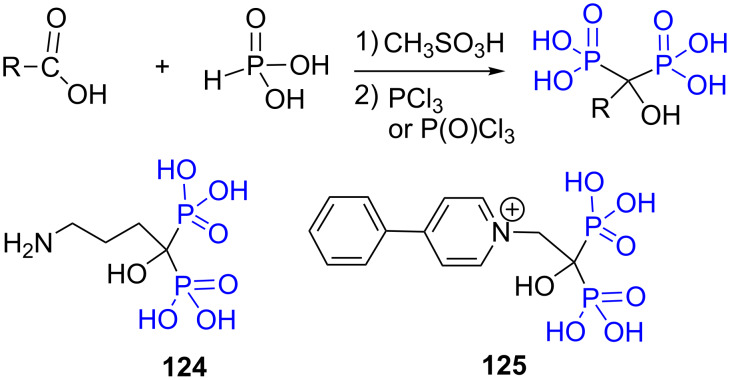
Reaction of carboxylic acid with phosphorous acid and examples of compounds prepared by this way.

The nitrile group can be replaced in this reaction with a carboxylic acid function. In that case the final product is a hydroxymethylenebis-phosphonic acid (Figure 34). This reaction was optimized to produce, for instance, compounds **124** [233–234] or **125** [229]. This reaction is not further detailed herein as it was recently reviewed [235].

### 7. Preparation of phosphonic acid by oxidation of phosphinic acid

**Figure 35:**
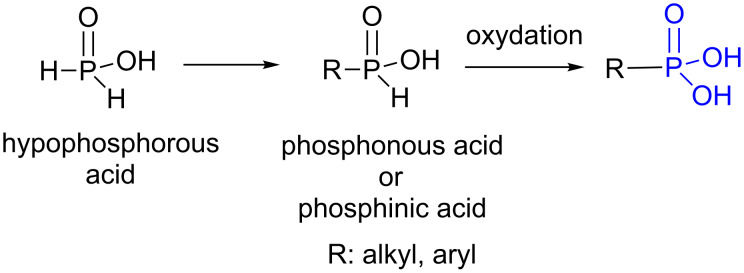
Synthesis of phosphonic acid by oxidation of phosphinic acid (also identified as phosphonous acid).

Phosphinic acid derivatives (also identified as phosphonous acid) are prepared by reaction of hypophosphorous acid (Figure 35) on alkene or alkyne (hydrophosphonation) [236], by its addition on aldehyde or imine [237] or by hydrolysis of alkyl or aryldichlorophosphine (RPCl_2_) [238] (for a review see reference [239]). Then, these phosphinic acids constitute a suitable precursor to produce phosphonic acids by oxidation (Figure 35).

**Figure 36:**
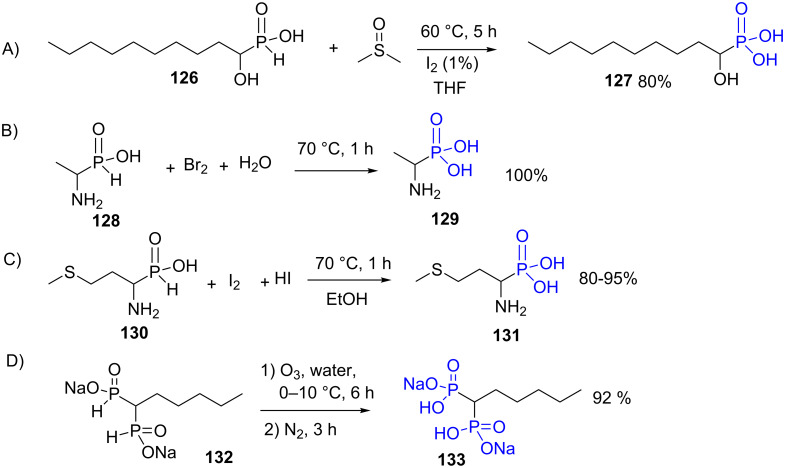
Selection of reaction conditions to prepare phosphonic acids from phosphinic acids.

The oxidation of phosphinic acid is readily achieved in the presence of DMSO [26] and a catalytic amount of iodide as exemplified by the synthesis of compound **127** by oxidation of **126** [240] (Figure 36A). This method was also applied to prepare 1-aryl-1-hydroxymethylphosphonic acid [241]. HgCl_2_ [242] or bromine water are also efficient for the oxidation of phosphinic acid. As an example, the phosphinic acid **128** (Figure 36B) which is prepared by the addition of hypophosphorous acid on imine, was converted quantitatively in α-amino phosphonic acid **129** with bromine water [243] (Figure 36B). HgCl_2_, despite its toxicity and environmental hazard is nevertheless an efficient reagent for the oxidation of phosphinic acid [244]. Diiodide in the presence of acid (HI) in water/ethanol solution yields the oxidation of the phosphinic acid **130** without oxidizing the thioether function [245] (Figure 36C). The Atherton–Todd conditions [246] (CCl_4_, NEt_3_, H_2_O) in the presence of water was also applied for the oxidation of phosphinic acid [247]. The oxidation of the phosphinic acid into phosphonic acid can be achieved with air or by a catalytic process involving the presence of palladium salts [236,248]. For instance, Kafarski et al. reported an efficient procedure that consisted in transforming the H-phosphinic acid into trivalent trimethylsilyl esters by reaction with hexamethyldisilazane followed by the oxidation with air followed by methanolysis [249]. A similar method that used a silylated intermediate and oxygen as oxidant was reported by Piettre et al. to produce alkyl-α,α-difluorophosphonic acid [250]. Ozone was also reported as oxidizing agent for the synthesis of phosphonic acid as exemplified by the synthesis of the diphosphonic acid **133** that was prepared from the phosphinic acid **132** [251] (Figure 36D).

### 8. Miscellaneous

If the most frequently used methods to prepare phosphonic acid have been reported above, others exist. In a non-exhaustive way of presentation, few of them are presented below.

**Figure 37:**
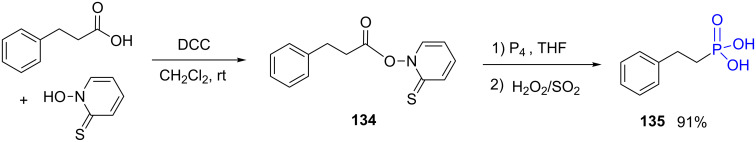
Synthesis of phosphonic acid from carboxylic acid and white phosphorus.

Barton et al. reported a procedure to prepare phosphonic acids from carboxylic acids that made use of white phosphorus (P_4_) [252]. First, the carboxylic acid is esterified with *N*-hydroxy-2-thiopyridone in the presence of DCC to produce **134** (Figure 37). This compound **134** was added to P_4_ solubilized in THF and was stirred for 30 minutes before replacing THF by DME. Then, H_2_O_2_ was added portion-wise and the solution was heated at reflux to produce the phosphonic acid **135**. For some compounds, the reaction with H_2_O_2_ occurred at rt and then SO_2_ is added to complete the oxidation reaction. The work-up is, however, tedious because some excess of P_4_ must be removed without any contact with oxygen. This procedure was applied to natural compounds including lipophilic carboxylic acid (e.g., linoleic acid) or amino acid (e.g., L-2-amino-4-phosphonobutyric acid).

**Figure 38:**
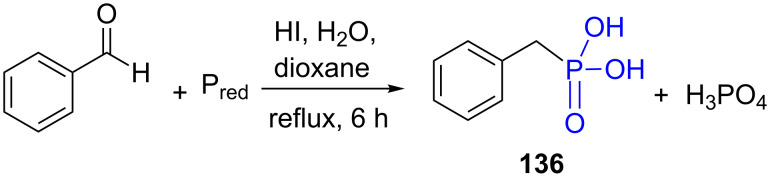
Synthesis of benzylphosphonic acid **136** from benzaldehyde and red phosphorus.

Red phosphorus is much easier to handle due to its polymeric nature that renders this compound much stable but also less reactive. Its reaction with benzaldehyde was reported to produce an α-hydroxy-phosphinic acid intermediate that was converted to phosphonic acid in the presence of HI in an aquous organic media at reflux (Figure 38). However, the benzylphosphonic acid (**136**) was formed simultaneously with phosphoric acid thus requiring a purification step [253].

**Figure 39:**
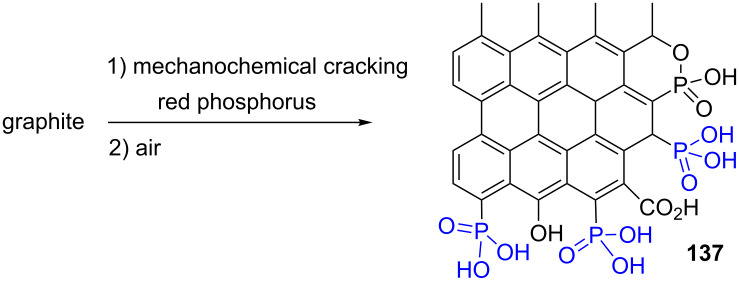
Synthesis of graphene phosphonic acid **137** from graphite and red phosphorus.

More recently, the phosphonation of graphite was reported. First, a mechanochemical cracking yielded carboradical intermediates that were reacted with red phosphorus and then oxidized in the presence of air to produce graphene phosphonic acid **137** [116] (Figure 39). This material which is water soluble was used as a non-toxic flame-retardant.

## Conclusion

Phosphonic acid is a functional group of interest for many current fields of research that include the development of bio-active compounds, medical imaging, material sciences or surface chemistry.

The most frequently used method to prepare phosphonic acids is stirring dialkyl phosphonates with concentrated HCl in aquous solution at reflux. Despite these drastic conditions, this protocol is a method of choice to prepare phosphonic acids that are stable within acidic media and that are thermally stable. The advantage of this methodology is that it can be applied on large scale and, as exemplified in this review, was applied to a huge variety of compounds. According to this method, the purification step is rendered simple by the fact that the excess of reagents and side products (alkyl halide) are easily removed under vacuum. For the molecules featuring acid sensitive functionalities, the McKenna’s method, that starts by the reaction of dialkyl phosphonate with bromotrimethylsilane to produce a bis-silylated phosphonate under mild conditions (usually at 20 °C), is a method of choice. This intermediate (silylated phosphonate) is then quantitatively converted into phosphonic acid by hydrolysis or methanolysis. The reaction has a very broad scope and the rare examples where side reactions were observed are actually due to the acidity generated by the final compound (phosphonic acid). According to the McKenna’s method the purification is also simple since the excess of reagent can be easily removed under vacuum.

Beside these two main methods to prepare phosphonic acids other direct methods can be selected. In that case the P–C bond is formed simultaneously with the production of a phosphonic acid functional group. In this way, the Moedritzer–Irani reaction is a well-documented and an efficient procedure. The limitation of this procedure can arise from the purification step which is mainly limited to crystallization.

Other methods are available as reported in this review; however, their use has a less broad scope but can be applied to prepare some specific phosphonic acids.
